# Optical mesoscopy, machine learning, and computational microscopy enable high information content diagnostic imaging of blood films

**DOI:** 10.1002/path.5738

**Published:** 2021-06-29

**Authors:** Michael Shaw, Rémy Claveau, Petru Manescu, Muna Elmi, Biobele J Brown, Ross Scrimgeour, Lisa S Kölln, Gail McConnell, Delmiro Fernandez‐Reyes

**Affiliations:** ^1^ Department of Computer Science, Faculty of Engineering Sciences University College London London UK; ^2^ Biometrology Group, National Physical Laboratory Teddington UK; ^3^ Department of Paediatrics College of Medicine of University of Ibadan, University College Hospital Ibadan Nigeria; ^4^ Department of Physics SUPA, University of Strathclyde Glasgow UK

**Keywords:** light microscopy, diagnostic imaging, supervised machine learning, haematologic tests, erythrocyte count, malaria, falciparum

## Abstract

Automated image‐based assessment of blood films has tremendous potential to support clinical haematology within overstretched healthcare systems. To achieve this, efficient and reliable digital capture of the rich diagnostic information contained within a blood film is a critical first step. However, this is often challenging, and in many cases entirely unfeasible, with the microscopes typically used in haematology due to the fundamental trade‐off between magnification and spatial resolution. To address this, we investigated three state‐of‐the‐art approaches to microscopic imaging of blood films which leverage recent advances in optical and computational imaging and analysis to increase the information capture capacity of the optical microscope: optical mesoscopy, which uses a giant microscope objective (Mesolens) to enable high‐resolution imaging at low magnification; Fourier ptychographic microscopy, a computational imaging method which relies on oblique illumination with a series of LEDs to capture high‐resolution information; and deep neural networks which can be trained to increase the quality of low magnification, low resolution images. We compare and contrast the performance of these techniques for blood film imaging for the exemplar case of Giemsa‐stained peripheral blood smears. Using computational image analysis and shape‐based object classification, we demonstrate their use for automated analysis of red blood cell morphology and visualization and detection of small blood‐borne parasites such as the malarial parasite *Plasmodium falciparum*. Our results demonstrate that these new methods greatly increase the information capturing capacity of the light microscope, with transformative potential for haematology and more generally across digital pathology. © 2021 The Authors. *The Journal of Pathology* published by John Wiley & Sons, Ltd. on behalf of The Pathological Society of Great Britain and Ireland.

## Introduction

Microscopic analysis of blood films is fundamental to many areas of haematology from research to clinical diagnosis [1]. Automated assessment of digitized blood films [2, 3] has potential to transform overstretched clinical services that require prompt and accurate assessment of large numbers of specimens. This need is particularly acute in low‐resource settings where human expert analysis of the blood film is the only tool available. Information‐rich blood film micrographs contain a wealth of details which allow classification and counting of blood cells and detection of blood‐borne parasites and bacterial infections. In contrast to alternative methods such as rapid diagnostic tests and flow cytometry, microscopy also allows visualization and analysis of cell morphology. However, the fundamental properties of light and practical optical engineering constraints limit the ability of a conventional light microscope to capture high‐resolution images with a large field of view (FoV), making it impossible to visualize an entire blood film at high spatial resolution in a single image. As a result, large images are often formed by sequential capture and subsequent stitching of multiple small image fields – a process which is slow and prone to subjectivity and inadequate sampling. In addition to the high cost of traditional, clinical grade whole slide imaging systems, many such devices are incapable of achieving the high spatial resolution often required for diagnostic image‐based blood film assays.

**Figure 1:**
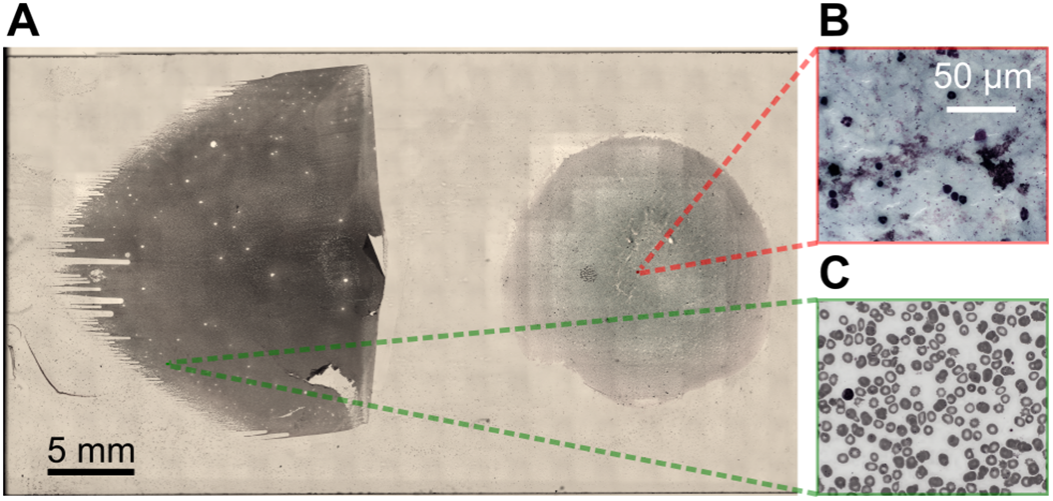
Brightfield microscopy image of Giemsa‐stained peripheral blood smears. (A) Overview image showing thin (left) and thick (right) films on a microscope slide, created by computational stitching of separate overlapping image fields captured using a 4×/0.16 objective lens. (B, C) Example of full field of view from within thick (B) and thin (C) films captured using a 100×/1.4 oil immersion objective. The spatial extent of the high‐resolution field of view is indicated by the small red and green boxes in the overview image on the left.

For illustration, consider the problem of imaging an entire thin blood film which lies within a rectangular patch on the microscope slide of 40 mm × 20 mm and has a thickness of approximately 3 μm. At modest spatial resolution, with a 20×/0.45 objective and a conventional large‐format scientific camera (2048 × 2048 6.5 μm pixels), the microscope has a FoV of 0.44 mm^2^ and a depth of field (DoF) of 3.7 μm. To capture the entire film would require approximately 1800 image fields in a single focal plane, or 3600 images over two focal planes (assuming a maximum separation of half the DoF) to fully sample the film axially. The problem is exacerbated at higher spatial resolution as magnification increases and DoF decreases with increasing numerical aperture (NA). In practice, diagnostic assays are typically based on the analysis of a small number of image fields, but the example demonstrates the practical difficulty of digitally capturing all the information within a blood film using a conventional optical microscope. Creating an extended FoV image by stitching together multiple small fields of view invariably results in artefacts due to spatial registration errors and brightness variations between image patches (Figure 1A). In recent years, a number of innovative techniques have been developed to increase the information capture capacity of the optical microscope, allowing high spatial resolution imaging with a large FoV. These approaches can be categorized as (1) purely optical – relying on novel optical and mechanical design and engineering; (2) computational imaging – optical encoding of additional sample information using novel hardware architectures followed by decoding using computational image processing; and (3) purely computational – increasing the information content of images post‐capture using prior knowledge about the sample and/or the imaging system. In this article we investigate three such approaches for microscopic imaging of blood films: (1) optical mesoscopy (OM) [4], in which a giant microscope objective lens combines low magnification with a high numerical aperture; (2) Fourier ptychographic microscopy (FPM) [5], in which high‐resolution information is captured using a low‐magnification objective lens via sequential illumination of the sample at a series of different angles; and (3) deep neural networks (DNNs), which leverage prior knowledge about sample structure and the correspondence between low‐ and high‐resolution images to increase image quality [6]. To assess the performance of these different methods for blood film imaging, we investigate their suitability for extraction of diagnostically relevant information, including red blood cell (RBC) morphology and the detection of small blood‐borne parasites such as the malaria parasite *Plasmodium falciparum*, from images of Giemsa‐stained peripheral blood films. The results are compared against images produced using the type of standard brightfield microscope commonly used for routine examination of blood films. We demonstrate the potential of the resulting large image datasets for automated analysis by developing simple image processing workflows for analysis and classification of RBC morphology. Finally, we discuss the potential for broader application and adoption of these novel methods in haematological imaging and beyond.

## Materials and methods

### Optical mesoscopy (OM) using a Mesolens

A Mesolens is a giant microscope objective lens designed for digital image acquisition (supplementary material, Figure S1A), which has a unique combination of low magnification (4×) and high numerical aperture (NA) (0.47) to allow sub‐cellular resolution imaging of sample volumes in excess of 100 mm^3^ [4, 7]. The lens is chromatically corrected across the entire visible spectrum, and multiple correction collars can be adjusted for imaging specimens with oil, glycerol, or water immersion. To capture the large, high‐resolution images produced by the Mesolens, the mesoscope system uses a chip‐shifting camera sensor (VNP‐29MC; Vieworks, Gyeonggi‐do, Republic of Korea) which records images by shifting a 29‐megapixel CCD chip. During acquisition, each camera pixel successively occupies nine positions in a 3 × 3 array. Subsequent reconstruction of each (260 megapixels, 506 Mb) takes approximately 5 s on a typical laboratory PC. The camera has a monochrome sensor and so colour brightfield images are created using a series of blue (445 GB 50; Comar Optics, Linton, Cambridge, UK), green (520 GB 50, Comar Optics), and red (610 GY 50, Comar Optics) coloured glass filters manually inserted into the illumination path between the white LED light source and the Mesolens. The three resultant colour channel images are then merged into a false colour RGB image and white‐balanced in Fiji [8] (supplementary material, Figure S1B,C). Prior to imaging, blood film slides were coated with immersion oil (Type LDF; Cargille, Cedar Grove, NJ, USA). Total image acquisition time was 540, 600, and 1440 ms for the red, green, and blue colour channel images, respectively.

### Fourier ptychographic microscopy (FPM)

FPM [5] is a wide‐field coherent imaging technique which exploits the fact that illuminating a thin sample at an oblique angle provides access to normally undetectable high spatial resolution information [9]. The method combines a large field of view with high spatial resolution, making it particularly attractive for imaging blood films, and it has previously been used for counting white blood cells [10] and high‐resolution imaging of infected red blood cells [11]. FPM relies on the capture of a series of images of the sample as it is illuminated sequentially by individual LEDs within a 2D array (supplementary material, Figure S2A). Combining the information contained within these images increases the effective numerical aperture from NA_obj_ to NA_syn_ = NA_obj_ + NA_ill_, where NA_ill_ is determined by the illumination from the LED furthest from the optical axis NA_ill_ = sin θ_max_. Extending the spatial frequency support in this way increases spatial resolution and improves visualization of fine structural details (supplementary material, Figures S2B and S3). We developed an upright FPM system [12] using a commercially available, low‐cost LED matrix (WS2812; WorldSemi, DongGuan, GuangDong, PR China) containing 22 × 22 RGB LEDs arranged on a square grid with an inter‐LED spacing of 7–8 mm. The LED matrix was mounted 50–90 mm below the sample (depending on the objective used) on a custom 3D‐printed holder which was imaged using an air immersion objective lens (4×/0.16, 10×/0.3 or 20×/0.45 – UPLSAPO4×, MPLFLN10×, and MPLFLN20× Olympus, Shinjuku City, Tokyo, Japan) and a tube lens with a focal length of 200 mm, giving a total system magnification of 4.4×, 11.1× or 22×. Images were recorded using a monochrome camera (IRIS 15; Teledyne Photometrics, Tucson, AZ, USA) with a sensor comprising 5056 × 2968 4.25 μm pixels, giving a field of view of 4.8 mm × 2.8 mm at 4.4×. Image capture was synchronized with the LED illumination sequence using a microcontroller (Uno; Arduino, Somerville, MA, USA). For each image set, the sample was sequentially illuminated with 225 individual LEDs arranged within a filled circle on the matrix. With a camera exposure time of 100 ms, the total acquisition time for each (monochrome) image was slightly less than 30 s. Colour images were captured by combining images acquired under illumination by red, green, and blue LEDs. Images were reconstructed using a version of the iterative phase retrieval method described by Tian *et al* [13] modified to reduce background‐related image artefacts [14].

### Image enhancement using a convolutional neural network (CNN)

Machine learning (ML)‐based computational image enhancement was performed using a convolutional neural network (CNN) with an encoder–decoder architecture consisting of three 2D convolutional layers (Conv2D), followed by nine residual layers (ResBlock), two 2D transposed convolutional layers (UpConv2D), and one 2D convolutional layer with a hyperbolic tangent (tanh) activation function at the end [15] (supplementary material, Figure S4). To generate training data, high‐resolution reference images of blood films captured with a 100×/1.4 oil immersion lens were degraded by convolution with theoretical intensity point spread functions (PSFs) for 10×/0.3 and 20×/0.45 objective lenses, where the values of the PSFs were evaluated using the Richards and Wolf 3D model [16] assuming monochromatic light at a wavelength of 610 nm. Each captured image field corresponded to an area of 166 × 142 μm. After convolution, simulated images were down‐sampled, by factors of 10 and 5, to account for differences in magnification. The model was then pre‐trained using a total 52 high‐resolution–simulated low‐resolution image pairs, each 2560 × 2160 pixels in size, before being fine‐tuned using a set of 22 real high‐resolution–low‐resolution image pairs. During each iteration, 512 × 512 patches were randomly cropped from each training image pair. Random rotations and flips were applied to further augment the training set. An Adam optimizer with an initial learning rate of 0.0003 was used to minimize the mean absolute error between the target (ground truth) and predicted images in both spatial and Fourier space. Model training was performed using Tensorflow's GPU implementation [17], which took approximately 18 h on an Intel (Santa Clara, CA, USA) Core i9 3.1 GHZ CPU with a NVIDIA (Santa Clara, CA, USA) GeForce RTX GPU with 12 Gb of memory.

The model was evaluated on unseen real image fields acquired with 10×/0.3 and 20×/0.45 objective lenses. Following training, subsequent processing of low‐resolution images took approximately 0.5 s per image field on a standard laboratory PC with a graphics processor.

### Conventional brightfield microscopy

For comparison with OM and FPM results, reference images of blood films were also acquired using a conventional motorized brightfield microscope (BX63, Olympus) with a 100×/1.4 oil immersion objective lens (MPlanApo N, Olympus) and a digital colour camera (Edge 5.5c; PCO, Kelheim, Lower Bavaria, Germany). Images were de‐mosaiced and then white‐balanced using a reference image of a blank microscope slide. Further colour balancing was performed manually in order to match the colour of RBCs to those in FPM and OM images of the same slides. To account for the shallow depth of field, a focal series (z‐stack) of images spanning the thickness of the blood film was captured for each region of interest in the sample. Each z‐stack was then processed using a wavelet‐based extended depth of field algorithm [18] to render a single image with all sample features in focus. For CNN training and testing, additional images were acquired using the same microscope with 10×/0.3 and 20×/0.45 (MPLFLN, Olympus) objective lenses.

### Sample collection and blood film preparation

The internationally recognized ethics committee at the Institute for Advanced Medical Research and Training (IMRAT) of the College of Medicine, University of Ibadan (COMUI) approved this research on the platform of the Childhood Malaria Research Group (CMRG) within the academic Department of Pediatrics, University of Ibadan, as well as at school and primary care centres throughout the city of Ibadan with permit numbers UI/EC/10/0130 and UI/EC/19/0110. Parents and/or guardians of study participants gave informed written consent in accordance with the World Medical Association ethical principles for research involving human subjects.

Blood films were prepared at the College of Medicine, University of Ibadan, Nigeria according to World Health Organization malaria microscopy standard operating procedures MM‐SOP‐01 to 06b. A 12‐μl and a 2‐μl droplet from a finger prick blood sample were deposited on different parts of a cleaned glass microscope slide. The larger droplet was then spread across a circular region of diameter 10 mm using a pipette tip to create a thick film and the smaller droplet was spread along the length of the slide using a second clean glass slide to form a thin film (Figure 1). Blood films were then fixed by dipping the thin film end of the slide into methanol for 2 s. After air drying, slides were coated with Giemsa solution (Merck, Darmstadt, Hesse, Germany) and left for 8–10 min before flushing away excess stain using buffered water. Slides were then air‐dried a second time before imaging.

### Analysis of RBC morphology

For OM, we developed a simple computational workflow to segment and analyse RBCs from thin blood films. Illumination nonuniformity was corrected using adaptive thresholding with a Gaussian kernel. A binary image mask was then generated using K‐means clustering‐based image segmentation [19] to partition image pixels into foreground (RBCs) and background classes. Holes in RBCs were removed by morphological filling, and a size exclusion threshold was then applied to each discrete binary object to remove overlapping RBCs and smaller objects from the binary image. A set of 45 shape descriptors for each binary object was then computed using the open‐source image analysis software CellProfiler [20]. For FPM, RBCs were segmented by applying a Sobel edge detector to the unwrapped FPM phase images, followed by global thresholding using Otsu's method [21], and finally morphological filling to create a set of binary RBC objects. As for OM images, 45 shape descriptors were then computed for each segmented RBC. Ninety‐three successfully segmented RBCs were manually classified as round or spiculated (echinocytes and acanthocytes) and the corresponding shape feature vectors were used to train a set of supervised machine learning classifiers using MATLAB's (Mathworks, Natick, MA, USA) Classification Learner app. Of the 25 different classifiers tested, a quadratic support vector machine gave the highest prediction accuracy (97.8%).

### Space bandwidth product as a measure of the information capture capacity of a microscope

The information capturing capacity of a microscope can be quantified by its space bandwidth product (SBP) [5], which is equal to the number of image pixels required to sample the full field of view (FOV), SBP = FOV/(0.5*r*)^2^. In the absence of imaging aberrations, the spatial resolution, *r*, is determined by the NA of the objective lens and the characteristics of the illumination. For brightfield microscopy with broadband illumination, the configuration used for the great majority of blood film imaging, the lateral resolution can be quantified using Abbe's resolution criterion, *R*_Abbe_ = *λ*/2NA. For simplicity, *λ* can be assumed to represent an average of the illumination power spectrum, the spectral transmittance of the microscope components, and the spectral responsivity of the camera. The achievable FOV is also dependent on the NA, as practical constraints to the diameter of the objective lens pupil means that focal length decreases with increasing NA and hence magnification increases. The NA also determines the depth of field of the microscope according to [22] DOF ~ *λn*/NA^2^ + *n∙e*/(*M*∙NA), where *n* is the refractive index of the objective lens immersion medium, *e* is the size of the camera's pixels, and *M* is the magnification of the microscope system. This means that high spatial resolution images have an inherently shallow depth of field as well as a small field of view.

## Results

### OM, FPM, and DNNs increase information content and spatial resolution of images for blood film analysis

**Figure 2:**
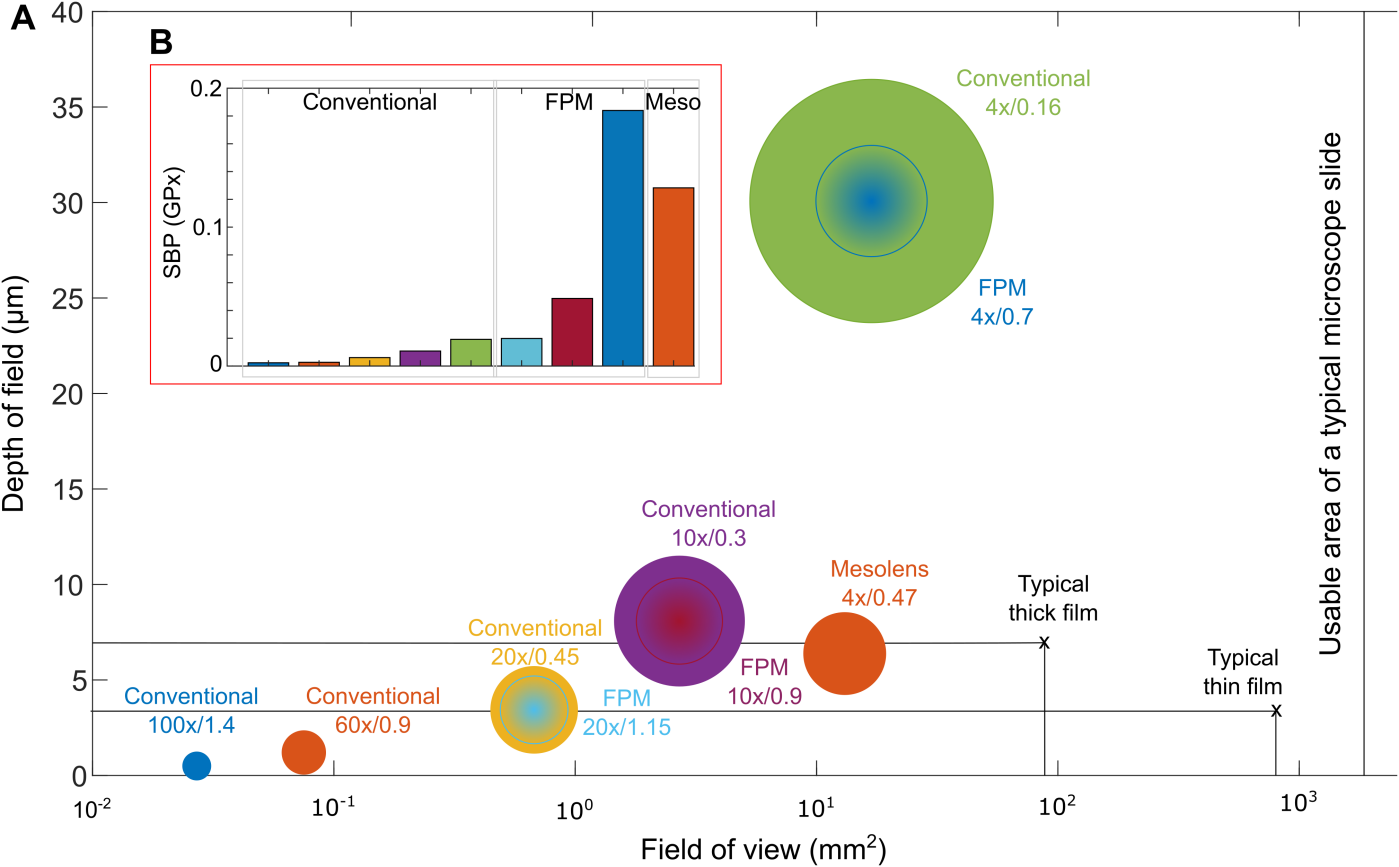
Field of view, depth of field, spatial resolution, and information capture capacity in optical microscopy. (A) Log‐linear scatterplot showing field of view and depth of field of conventional brightfield microscopy, FPM, and Mesolens systems. The diameter of the filled circles is proportional to the lateral spatial resolution of each system. FPM generates a complex image in which the effective lateral resolution depends on the properties of the object and in this case, the diameter of the circle represents the reciprocal of the coherent cutoff frequency (λ/NA). (B) SBP (a measure of information capturing capacity) of the different techniques in gigapixels.

The interdependence of the magnification and NA of the objective lens in a conventional microscope means that the capture of a larger imaging volume is necessarily achieved at the expense of spatial resolution. Figure 2A shows the decrease in lateral spatial resolution with increasing FoV and DoF for five common objective lenses. The figure inset (Figure 2B) illustrates how the image information content, quantified using the space‐bandwidth product, also decreases with increasing spatial resolution. In practice, this means that conventional high‐resolution images inherently carry less information than their low‐resolution, larger FoV equivalents. Both FPM and OM deviate from this trend. FPM increases lateral spatial resolution (effective NA) whilst maintaining FoV. As the effective NA (NA_syn_) increases, reconstructed FPM images have a correspondingly shallower DoF [22]; however, recovery of the full complex optical field in FPM enables images to be computationally refocused post‐capture to visualize the sample over the full DoF of the (low NA) objective lens [12]. In OM, an objective lens with a long effective focal length (low magnification) and a high NA enables capture of high spatial images with a substantially larger field of view (6 mm in diameter) than is possible with a conventional microscope.

The typical size and thickness of thick and thin films are indicated by the black crosses in Figure 2A, where the thick film is assumed to lie within a circular region of diameter 10 mm and the thin film within a rectangular patch 40 mm × 20 mm. For all sample features to be simultaneously in focus, the DoF must exceed the thickness of the blood film. For thick films, this is the case for a typical 4× (NA = 0.16) or 10× (NA = 0.3) objective. For thin films, both the Mesolens (NA = 0.47) and a typical 20× (NA = 0.45) objective also have a sufficient DoF. The large area of a typical blood film means that a low‐magnification objective is required in order to capture a significant fraction of the film in a single image. A microscope with a large format (21.49 mm × 12.61 mm) camera and a 4× objective has an FoV of 16.9 mm^2^, equivalent to 21.6% of the area of a thick film or 2.1% of the area of a thin film. The Mesolens employs a sensor‐shifting camera to allow a similar FoV of 13.1 mm^2^, equivalent to 16.6% of the area of a thick film or 1.6% of the area of a thin film. More importantly, whilst a conventional microscope with a 4×/0.16 objective lens has a lateral resolution of only around 2.3 μm, at the same magnification the OM and FPM systems achieve sub‐μm lateral resolution which is sufficient to resolve important details such as the fine structure of blood cells.

**Figure 3:**
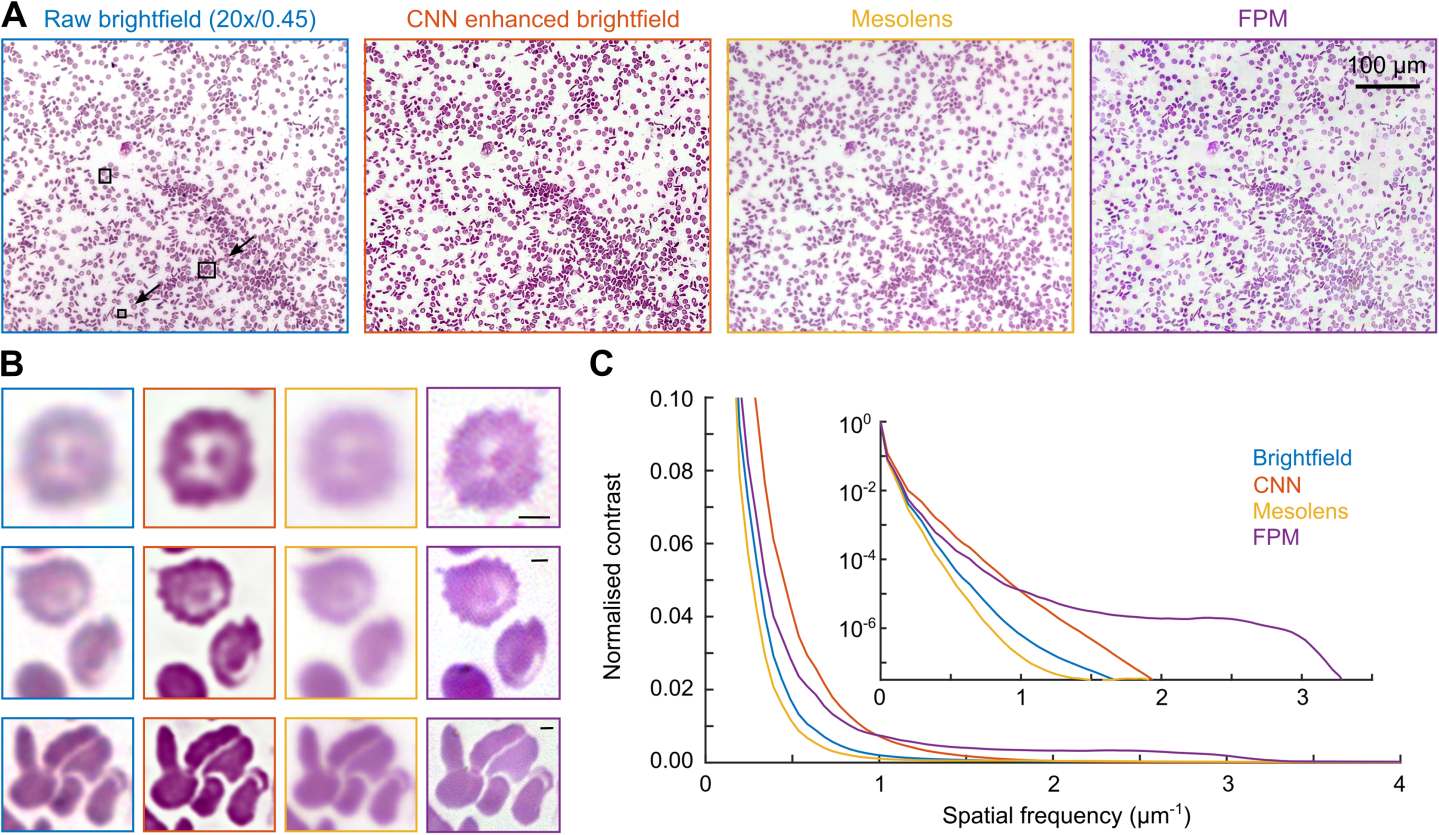
Comparison of images produced using different methods. (A) 0.5 mm × 0.3 mm region of interest taken from images of a Giemsa‐stained blood film. (B) Magnified views of the boxed regions shown in A; scale bars are 2 μm. All images were self white‐balanced to facilitate comparison. (C) Measured power spectrum (magnitude of a fast Fourier transform) for the green channel of the images in A illustrating differences in contrast and the extent of the spatial frequency support for the different methods.

To compare the performance of the different methods, the same thin blood film was imaged using FPM, OM, and a conventional microscope system, with the latter image also processed using the previously described trained CNN model. In all cases, the nominal spatial resolution of the raw images was approximately the same, as the NA of the objective lens in each system was between 0.45 and 0.47. Qualitative assessment of the image results (Figure 3A,B) reveals several interesting features. Firstly, images produced by the Mesolens and a conventional microscope are similar; that the conventional image is slightly sharper is likely due to a small tilt of the sample with respect to the focal plane of the Mesolens. The effect of the CNN is primarily to increase image contrast and sharpen the edges of the RBCs. Whilst this edge enhancement allows clearer separation of RBCs in regions where they are densely clustered (bottom row of Figure 3B), visualization of fine morphological details, such as the membrane projections of the spiculated RBCs in the top and middle rows of Figure 3B, is only possible in the high‐resolution FPM image reconstructions. These differences are reflected in the radial power spectrum of the images shown in Figure 3C, where it can be seen that the effect of the CNN is to increase the contrast at intermediate to high spatial frequencies without significantly extending the spatial frequency cutoff beyond the diffraction limited value of ~1.7 μm^−1^. The capture of high frequency information in FPM both increases contrast for high spatial frequencies and extends the support of the optical transfer function (the maximum spatial frequency captured by the microscope) with a corresponding increase in image resolution (supplementary material, Figure S2B).

### OM and FPM enable quantitative analysis of RBC morphology over large areas of the blood film

**Figure 4:**
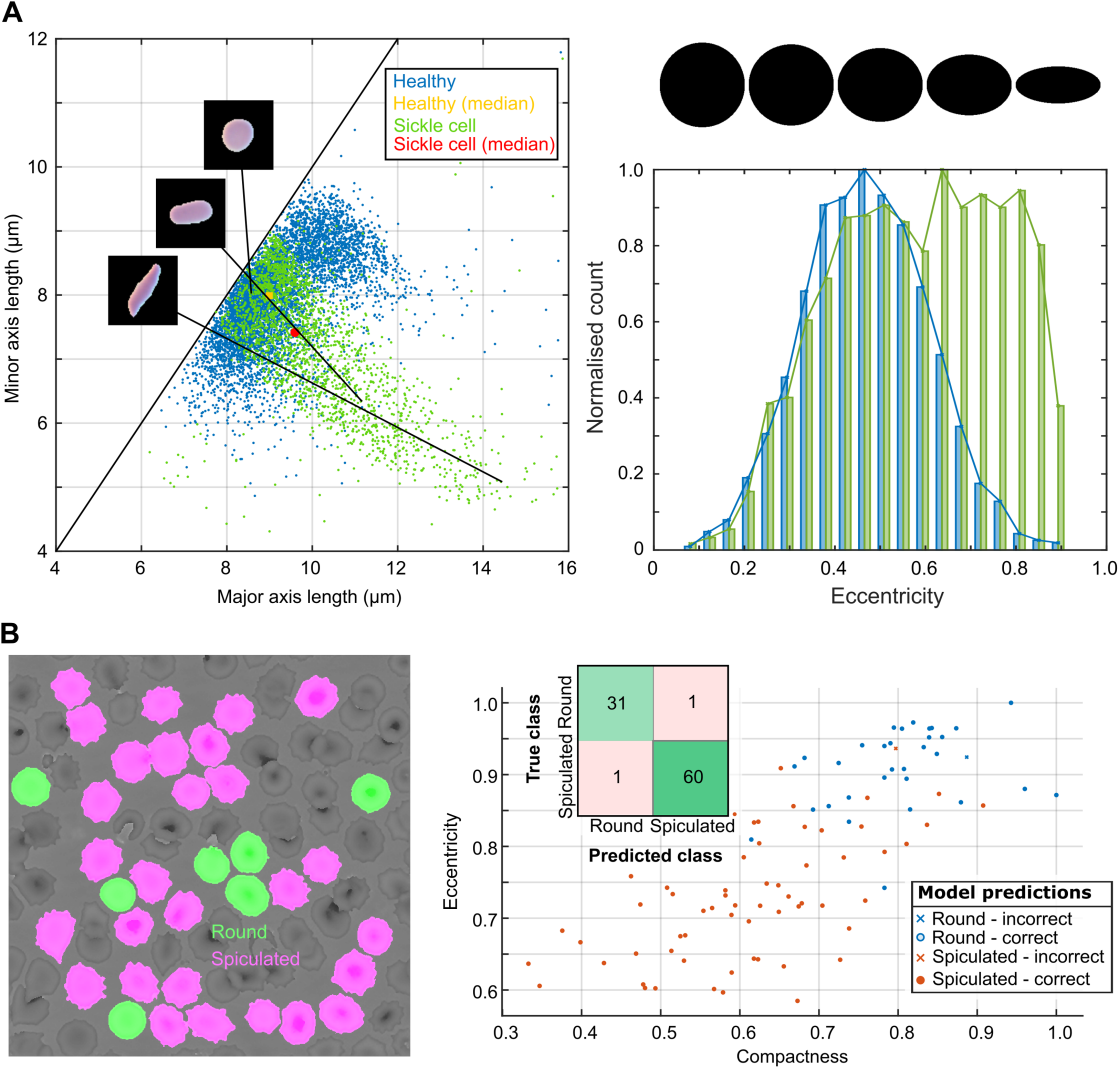
Measurement of RBC morphology from OM and FPM images of thin films. (A) Comparison of RBC morphologies measured from OM images of peripheral blood smears from a healthy patient (blue) and a patient diagnosed with sickle cell (HbSS) (green). Analysis performed on all segmented cells within a 1.95 mm × 1.95 mm field of view in a thin film from each patient. For the healthy patient, this corresponds to 6166 segmented cells and for the HbSS patient, 2281 cells. Left: scatter plot showing length of major and minor axes of ellipses fit to segmented RBCs. Right: normalized histograms showing measured eccentricity of segmented RBCs. (B) Classification of RBCs from FPM images. Left: segmented RBCs are manually classified as round (green) or spiculated (pink). Right: scatter plot showing compactness and eccentricity of classified RBCs used in the training set. The inset shows the confusion matrix for RBCs classified using a quadratic support vector machine. Overall accuracy is 97.8%.

Variations in RBC morphology provide important diagnostic cues [23]. Whilst conventional microscopic techniques offer sufficient spatial resolution to detect subtle morphological differences, their FoV is typically too small to capture enough RBCs for detection of rare phenotypes and extraction of robust population‐wide statistics. To investigate the suitability of OM and FPM for morphological assessment, we developed simple illustrative computational workflows to segment and classify RBCs from images of thin blood films (Figure 4).

For OM, we analysed differences in RBC morphology for a patient diagnosed with sickle cell disease (HbSS) and a healthy non‐sickle (HbAA) control. The left panel of Figure 4A shows a scatter plot of the minor and major axes of segmented RBCs, 6166 cells for the healthy control and 2281 cells for the HbSS patient. For the control, most RBCs are near circular and lie close to the diagonal (major‐axis length = minor‐axis length). The elongation of RBCs for the HbSS patient results in a qualitatively different distribution characterized by a large number of (off‐diagonal) highly elliptical RBCs. The right panel of Figure 4A shows the same data plotted as an ellipse eccentricity (*e*, the ratio of the distance between the foci of the ellipse and its major axis length) histogram. For the control, RBC eccentricity follows a normal distribution centred at *e* ~ 0.5. For the HbSS patient, the histogram is bimodal with a distribution that can be approximated as a sum of two Gaussians (non‐linear least squares fit, *R* ^2^ = 0.97) with mean and standard deviations (SDs) of 0.49 and 0.16 and 0.81 and 0.12. The first of these terms closely matches the eccentricity distribution for the control (mean = 0.47, SD = 0.14, *R* ^2^ = 0.996), suggesting that the second Gaussian term describes aberrant RBC morphologies associated with HbSS. This analysis was performed for a patch size of 1.95 mm × 1.95 mm. Assuming the same RBC number density across the full (13.1 mm^2^) Mesolens FoV, the analysis would include almost 21 200 RBCs for the control sample and more than 7800 RBCs for the HbSS patient.

The higher spatial resolution of FPM allows the visualization and assessment of more subtle differences in RBC morphology. As an example, we segmented and classified RBCs in FPM images of a thin blood film captured with a 10×/0.3 objective as spiculated or round based on the presence or absence of characteristic spiky membrane projections. The spiculated class includes both echinocytes (regularly spiculated) and acanthocytes (irregularly spiculated), where the latter can indicate a variety of disorders including liver disease, anaemias, and some hereditary conditions. Both classes cluster reasonably well by eccentricity and compactness (defined as mean squared distance of the object pixels from the centroid normalized by the area). The number of cells that can be analysed in this way is limited primarily by the reliability of the cell segmentation, which is strongly dependent on the RBC density and the proportion of overlapping or touching cells. For Figure 4B, we were able to segment and analyse 35 RBCs in a 76.5 μm × 76.5 μm patch. Assuming a similar RBC density and segmentation rate, this equates to the detection and classification of ~13 000 RBCs over the entire (10×) FPM FoV.

### FPM and ML improve visualization of *Plasmodium falciparum* parasites

**Figure 5:**
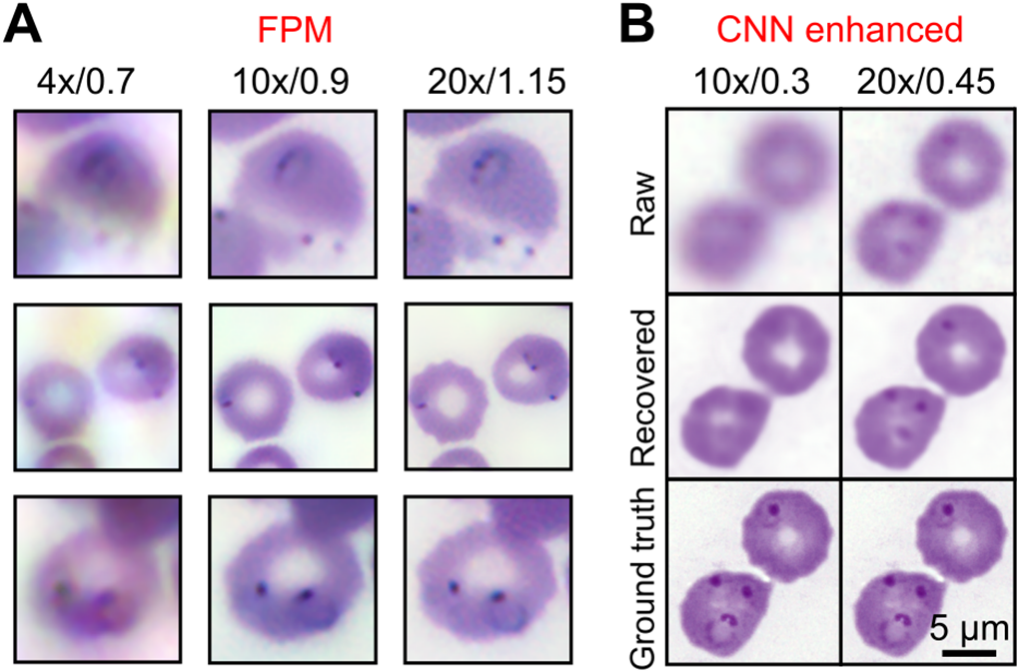
Parasite detection in FPM and CNN enhanced images of Giemsa‐stained thin blood films. (A) FPM amplitude images showing *P. falciparum*‐containing RBCs captured using different objective lenses. The NA indicated corresponds to the final reconstructed image. (B) Raw, CNN recovered, and ground truth images of *P. falciparum*‐containing RBCs captured using 10×/0.25 and 20×/0.45 objective lenses. Ground truth conventional brightfield images captured using a 100×/1.4 oil immersion objective lens.

The small size of many blood‐borne parasites and the presence of morphologically similar ‘distractors’, arising from non‐specific staining or contamination, necessitate high‐resolution imaging for accurate parasite detection in blood films. To assess the suitability of FPM and CNNs for parasite detection, we visually examined images of Giemsa‐stained thin films clinically assessed as positive for the *P. falciparum* malaria parasite (MP). *P. falciparum* typically presents as small ring‐shaped objects, typically ~2 μm in diameter, comprising a dark, densely stained chromatin spot surrounded by a fainter cytoplasmic ring. Figure 5A shows FPM images of the same three regions within a thin film captured using different objective lenses to illustrate how the NA of the objective and the resulting synthetic NA of the reconstructed image affect the visibility of MPs (for the corresponding full images, see supplementary material, Figure S3). In the 4×/0.7 images, although parasites are visible with sufficient contrast to pick out the chromatin spot, the spatial resolution is inadequate to clearly visualize the ring structure. For many samples, which are often sub‐optimally prepared, we find that such images are often inadequate for reliable parasite identification. By contrast, MPs are clearly resolved in FPM images captured with 10× and 20× objectives. The 20× images with a synthetic NA of 1.15, in particular, compare well with conventional brightfield images captured using a 100×/1.4 objective shown in the lower row of Figure 5B. Based on this, we anticipate a higher MP detection efficiency (accuracy) for 20×/1.15 images; however, this comes at the expense of a smaller field of view and resulting number of RBCs. As a result, the sensitivity for detection of a single MP may not necessarily be higher for higher‐magnification images.

Figure 5B shows the effect of the CNN on images of RBCs containing MPs (for the corresponding full images field, see supplementary material, Figure S5). In this case, the network was trained using a set of image fields of a single slide captured using 100×/1.4, 20×/0.45, and 10×/0.3 objective lenses and then used to estimate 100×/1.4 images from lower‐resolution images of different fields within the same slide. As noted previously (Figure 3), the CNN has the effect of sharpening images, with RBC membranes in 10× and 20× images better defined in estimated high‐resolution images. Quantitatively, the normalized variance (a measure of image sharpness) increases from 61.2% to 83.9% of the value for the 100× ground truth image for images captured with the 10× objective and from 65.6% to 90.7% for 20× images. The CNN also increases the structural similarity index with the ground truth image from 0.70 to 0.77 and from 0.73 to 0.76 for the 10× and 20× images, respectively. However, we find that the CNN is unable to render the four MPs visible from the 10× image. In the 20× image, the sharpening effect increases the contrast of the chromatin spot but fails to reveal significant additional parasite structure. This suggests that the CNN is unlikely to improve the visualization of MPs in low magnification (low‐resolution) images which are not already, to some extent, visible in the raw data. However, by increasing image sharpness/contrast, the network may aid the (manual or automated) detection of MPs.

## Discussion

To inform relevant clinical pathways, image‐based blood film analysis requires sufficient spatial resolution to detect (often subtle) morphological features and small objects, and a suitably large FoV to capture enough cells, or other objects of clinical interest, for statistical robustness. Our results show that OM, FPM, and CNNs can all be applied to provide an increased capacity for extraction of diagnostically important information of blood films. Although our analysis has been restricted to RBCs, in particular assessment of morphology and the detection of intracellular parasites, we note that the three methods can also be applied to imaging of WBCs (supplementary material, Figure S6). As with RBCs, we anticipate that combining a large FoV with high spatial resolution offers significant potential for improved image‐based diagnostic assays, such as the WBC differential count and the identification of malignant WBCs through their aberrant morphology.

Although our analysis has been restricted to Giemsa‐stained thin films, the methods are directly applicable to other stains and preparation protocols. Researchers have also effectively applied FPM for high‐resolution imaging of stained tissue sections [24]. By capturing sample phase information, FPM holds potential for label‐free structural imaging, albeit at the expense of the specificity provided by chemical staining. Thicker, scattering objects can present challenges for FPM; however, recent work [25, 26] has shown that modification of the image reconstruction algorithm can allow effective 3D FPM imaging. OM and CNN image enhancement methods are directly applicable to imaging and analysis of thicker samples such as thick blood films and tissue sections.

As with any new technology, the wider adoption of these methods depends on several factors, principally cost and complexity. FPM is relatively cheap and simple to implement on many of the microscopes currently in use for haematological research and clinical practice, requiring only the addition of a low‐cost LED array, a digital camera, and some off‐the‐shelf electronic components. However, care is required when acquiring and reconstructing images in order to avoid artefacts. In principle, FPM could also be employed to enhance the performance of other low‐cost automated microscope platforms [27, 28], allowing improved diagnostic imaging in resource constrained settings. Reconstruction of FPM images using common iterative phase retrieval algorithms can be relatively time‐consuming (several hours for the full FoV of a large format camera). Alternative approaches based on machine learning have shown promise in reducing the computational burden [29, 30] and we have obtained encouraging results using such methods for fast reconstruction of FPM images of blood films. Using a CNN‐based FPM reconstruction model trained using TensorFlow, we were able to obtain a high‐resolution full FoV image in approximately 3 min.

Aside from initial system setup and alignment, practical operation of the Mesolens is similar to a conventional brightfield microscope system, albeit with an increased sensitivity to any tilt of the specimen with respect to the focal plane of the objective lens because of the large FoV. Handling the resulting image datasets can require significant computational resources: an RGB OM image is around 1.5 Gb. Also, to date, there are only a small number of Mesolens systems housed in specialist academic research laboratories within the UK.

Machine learning methods are ubiquitous across many areas of science, technology, and medicine. At present, implementation often relies on expert computer scientists and on the availability of large amounts of training data. However, once the CNN model has been trained and validated, estimation of high‐quality, high‐resolution images from lower‐quality, low‐resolution image input is simple, fast, and requires only modest computer hardware.

Advances in computing hardware and the development of increasingly sophisticated computational analysis tools have created an increased capacity for storage and analysis of biological and biomedical image data. Our ability to interrogate biological systems, diagnose disease, and develop new therapeutic treatments may ultimately be limited by our ability to acquire suitable image data. Having demonstrated the capacity of OM, CNNs, and FPM to extract more of the rich structural information contained within from blood films, we look forward to their wider application for diagnostic imaging in haematology and more widely throughout digital pathology.

## Author contributions statement

MS and DF‐R designed the study. BJB and DF‐R carried out patient recruitment and diagnosis, and clinical sample collection and preparation. GM, LSK and RS captured and reconstructed Mesolens image data. MS, RC, ME and PM acquired and analysed brightfield microscope images. MS and RC designed and built the FPM system and captured and analysed FPM images. PM developed the CNN model and performed machine learning experiments and analysis. All the authors contributed to the analysis and interpretation of results. MS, DF‐R, RC and PM wrote the manuscript with input from all co‐authors. All the authors reviewed and approved the manuscript before publication.

## Supporting information


**Figure S1.** Photograph of the Mesolens and a typical thin blood film image
**Figure S2.** The principles of FPM and typical thin blood film images
**Figure S3.** FPM blood film images captured using different objective lenses
**Figure S4.** Schematic diagram illustrating the architecture of the CNN trained to enhance blood film images
**Figure S5.** Improving images of a Giemsa‐stained thin blood film using deep learning
**Figure S6.** Imaging WBCs in Giemsa‐stained thin blood films using OM, CNN, and FPM techniques

## Data Availability

The datasets used and analysed in this study are available from the corresponding authors on reasonable request.
