# Supplementary material for: Optical mesoscopy, machine learning, and computational microscopy enable high information content diagnostic imaging of blood films
Source: J Pathol. 2021 Jun 29;255(1):62–71. doi: 10.1002/path.5738 (PMC12086746; doi:10.1002/path.5738)
Supplement: Supplementary file 1 — Figure S1. Photograph of the Mesolens and a typical thin blood film image Figure S2. The principles of FPM and typical thin blood film images Figure S3. FPM blood film images captured using different objective lenses Figure S4. Schematic diagram illustrating the architecture of the CNN trained to enhance blood film images Figure S5. Improving images of a Giemsa‐stained thin blood film using deep learning Figure S6. Imaging WBCs in Giemsa‐stained thin blood films using OM, CNN, and FPM techniques [file PATH-255-62-s001.pdf]

Optical mesoscopy, machine learning, and computational microscopy enable high information content diagnostic imaging of blood films
M Shaw *et al.* *J Pathol* DOI: 10.1002/path. 5738

Supplementary Figures S1-S6

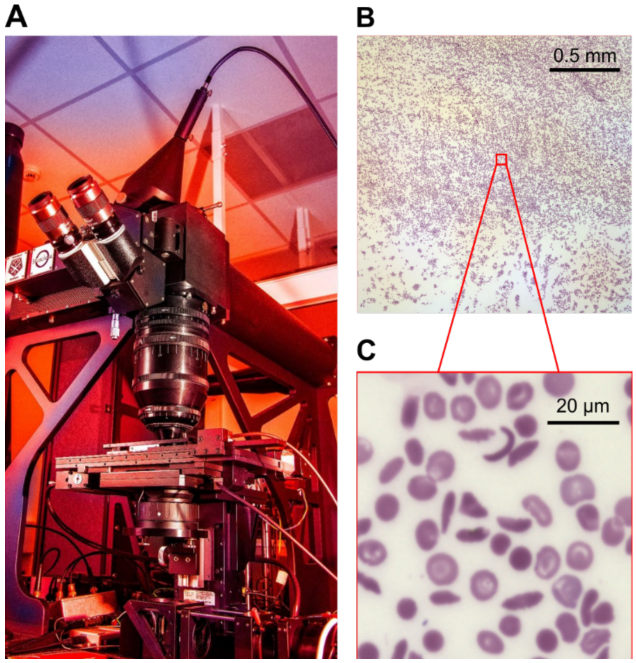

Figure S1. Photograph of the Mesolens and a typical thin blood film image.

(A) Photograph of the Mesolens microscope system. A white LED is used for brightfield illumination; coloured filters are inserted into the optical path to allow capture of red, green and blue images which are combined to create a false-colour merged composite image. (B) Typical OM image of a Giemsa-stained thin blood film. (C) Zoomed in view of the boxed region of interest in (B).

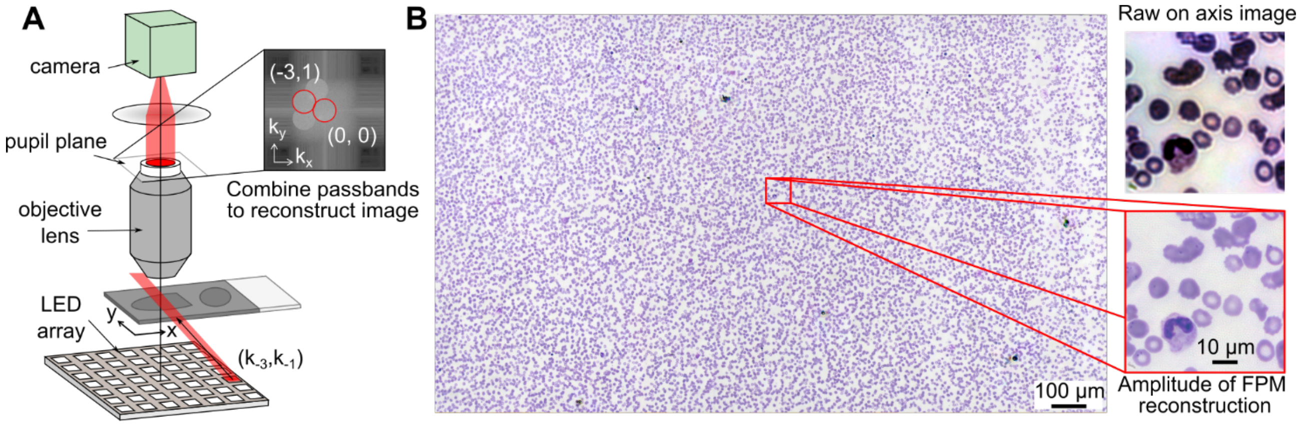

Figure S2. **The principles of FPM and typical thin blood film images.**

(A) Schematic diagram illustrating data capture and image reconstruction in FPM. A series of raw images are captured under sequential illumination with individual LEDs in a 2D array synchronized with the camera exposure. Each of these raw images contains a portion of the sample spectrum corresponding to the passband of the microscope objective shifted by an amount of determined by the illumination angle. High resolution complex images are reconstructed by sequentially adding information from each raw image in Fourier space using iterative phase retrieval. (B) Example FPM amplitude image of a Giemsa-stained thin blood film captured using a 10x/0.3 objective lens. Boxed images show zoomed in view of the reconstructed (bottom) and raw on axis (top) images of the area highlighted by the red square.

.

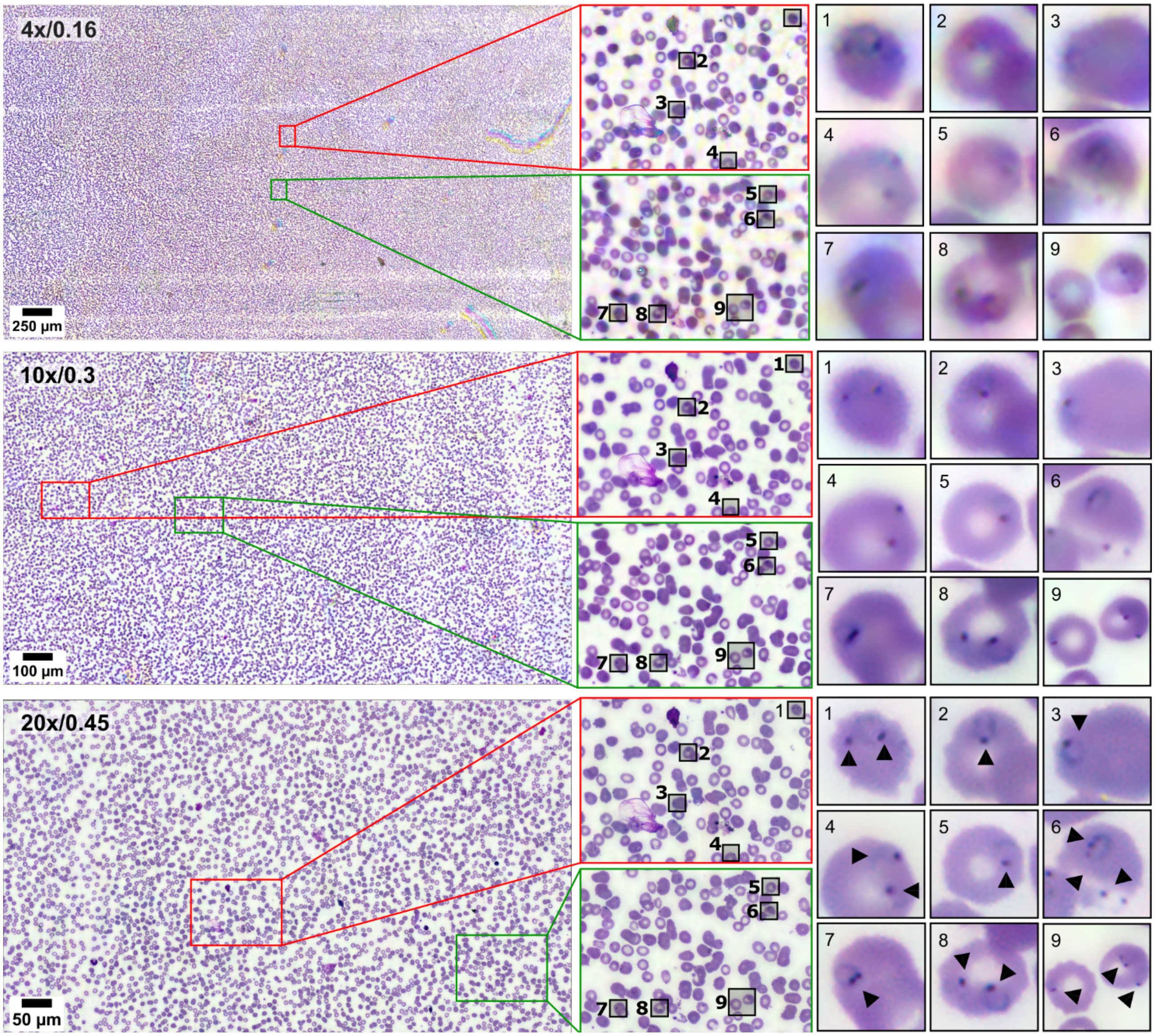

Figure S3. FPM blood film images captured using different objective lenses.

Images show the same area of a Giemsa-stained thin film prepared using a peripheral blood sample taken from a malaria positive patient, illustrating the potential for RBC visualization and parasite detection at different magnifications.

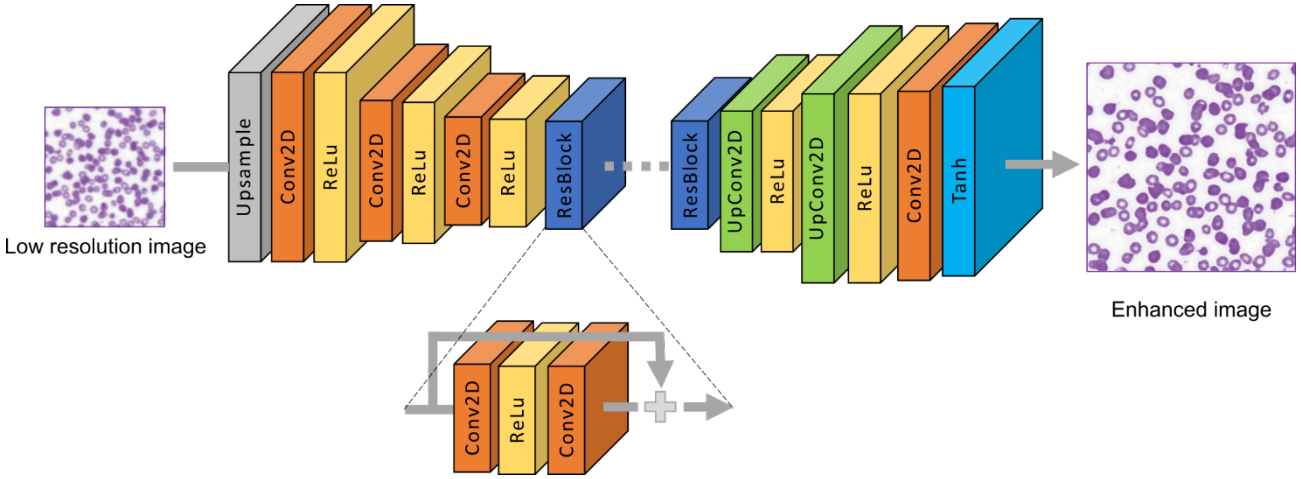

Figure S4. Schematic diagram illustrating the architecture of the CNN trained to enhance blood film images.

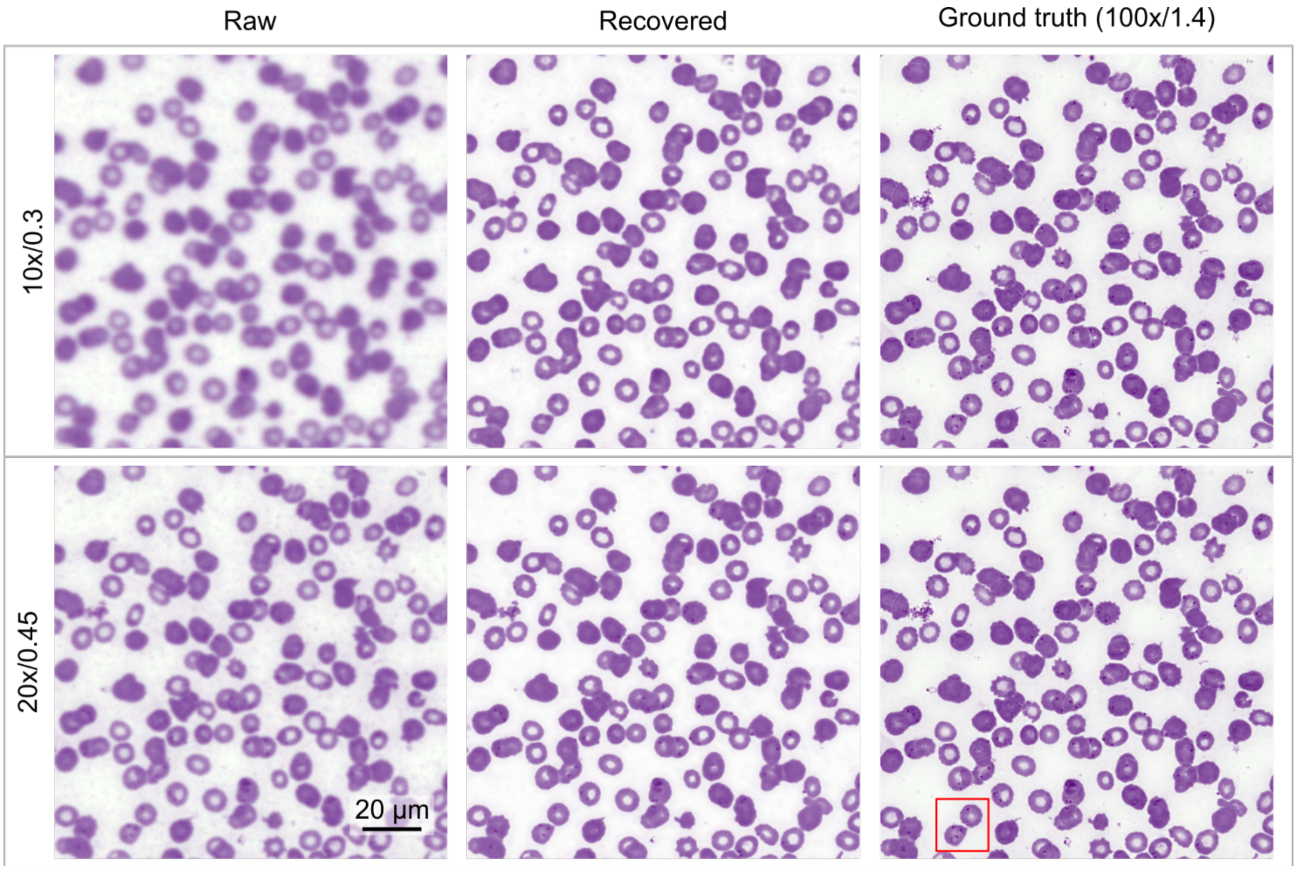

Figure S5. Improving images of a Giemsa-stained thin blood film using deep learning.

Left column shows raw images captured using 10x/0.25 and 20x/0.5 objective lenses. Centre column shows corresponding images recovered using a CNN trained using both simulated and real image data. Right column shows the same area of the blood film captured using a 100x/1.4 objective lens. Red box shows region of interest included in Figure 5 of the article.

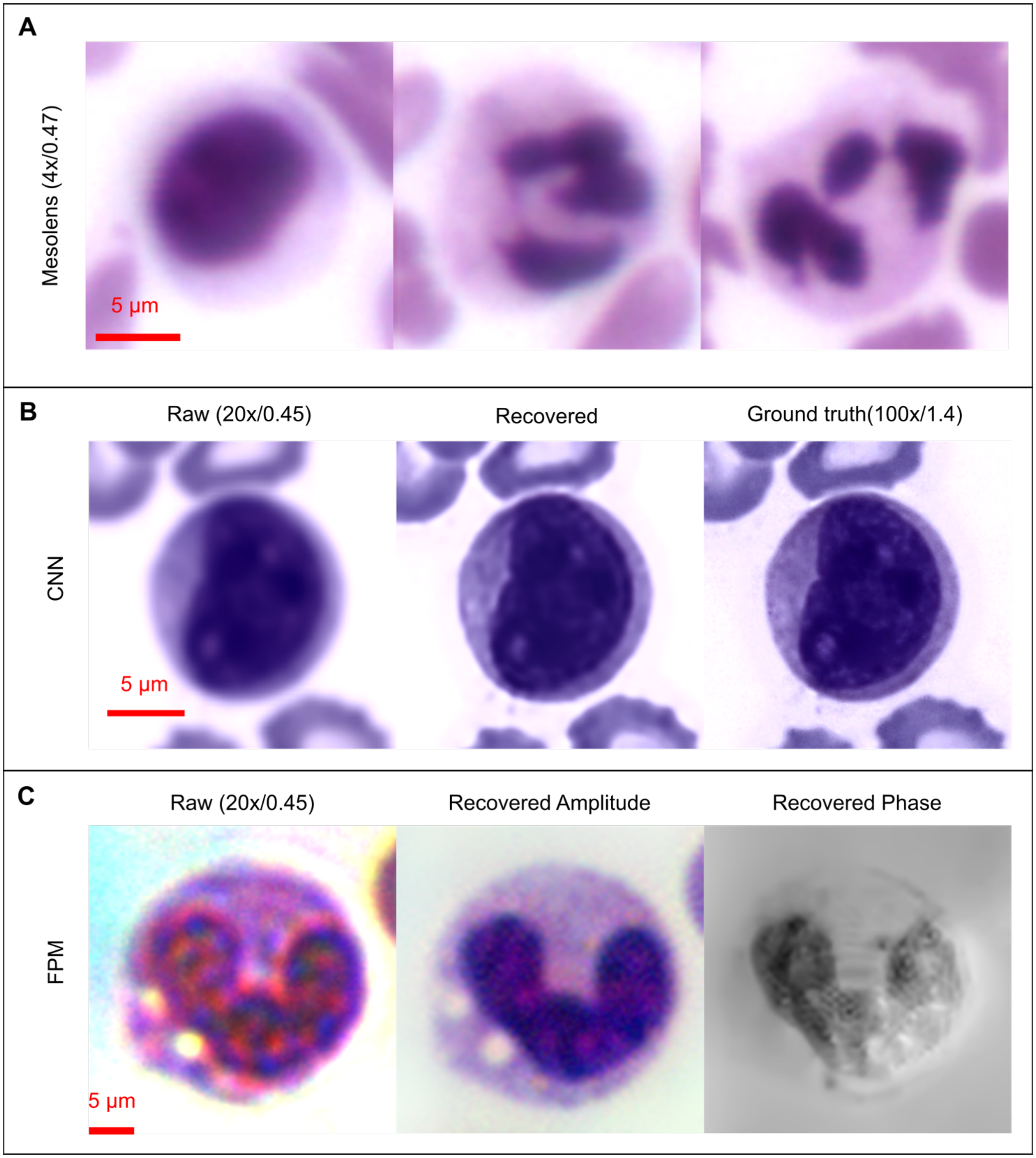

Figure S6. Imaging WBCs in Giemsa-stained thin blood films using OM, CNN and FPM techniques.

(A) WBCs cropped from single OM image. (B) Raw image (20x/0.45), CNN recovered image and corresponding ground truth image of a single WBC. (C) Raw on axis amplitude image, FPM reconstructed amplitude and phase images of a WBC.
